# Formation of diploid and triploid hybrid groupers (hybridization of *Epinephelus coioides* ♀ × *Epinephelus lanceolatus* ♂) and their 5S gene analysis

**DOI:** 10.1186/s12863-016-0443-9

**Published:** 2016-10-07

**Authors:** Wen Huang, Qinbo Qin, Huirong Yang, Shuisheng Li, Chaoqun Hu, Yude Wang, Yong Zhang, Shaojun Liu, Haoran Lin

**Affiliations:** 1 State Key Laboratory of Biocontrol, Institute of Aquatic Economic Animals, and the Guangdong Province Key Laboratory for Aquatic Economic Animals, Sun Yat-Sen University, Guangzhou, 510275 China; 2 Key Laboratory of Tropical Marine Bio-resources and Ecology (LMB) and Guangdong Provincial Key Laboratory of Applied Marine Biology (LAMB), South China Sea Institute of Oceanology, Chinese Academy of Sciences, Guangzhou, 510301 China; 3 Key Laboratory of Protein Chemistry and Developmental Biology of the State Education Ministry of China, College of Life Sciences, Hunan Normal University, Changsha, 410081 China; 4 College of Animal Science, South China Agricultural University, Guangzhou, 510642 China; 5 South China Sea Bio-Resource Exploitation and Utilization Collaborative Innovation Center, Guangzhou, 510275 China

**Keywords:** Natural polyploidization, Embryo development, Grouper, 5S gene, Heterosis

## Abstract

**Background:**

Interspecies hybridization is widely used to achieve heterosis or hybrid vigor, which has been observed and harnessed by breeders for centuries. Natural allopolyploid hybrids generally exhibit more superior heterosis than both the diploid progenies and their parental species. However, polyploid formation processes have been long ignored, the genetic basis of heterosis in polyploids remains elusive.

**Results:**

In the present study, triploid hybrids had been demonstrated to contain two sets of chromosomes from mother species and one set from father species. Cellular polyploidization process in the embryos had been traced. The triploid hybrids might be formed by failure formation of the second polarized genome during the second meiosis stage. Four spindle centers were observed in anaphase stage of the first cell division. Three spindle centers were observed in side of cell plate after the first cell division.

The 5S rDNA genes of four types of groupers were cloned and analyzed. The diploid and triploid hybrids had been proved to contain the tandem chimera structures which were recombined by maternal and paternal monomer units. The results indicated that genome re-fusion had occurred in the hybrid progenies.

To further elucidate the genetic patterns of diploid and triploid hybrids, fluorescence chromosome location had been carried out, maternal 5S gene (M-386) were used as the probe. The triploid hybrids contained fewer fluorescence loci numbers than the maternal species. The results indicated that participation of paternal 5S gene in the triploid hybrid genome had degraded the match rates of M-386 probe.

**Conclusions:**

Our study is the first to investigate the cellular formation processes of natural allopolyploids in hybrid fish, the cellular polyploidization process may be caused by failure formation of the second polarized genome during the meiosis, and our results will provide the molecular basis of hybrid vigor in interspecies hybridization.

**Electronic supplementary material:**

The online version of this article (doi:10.1186/s12863-016-0443-9) contains supplementary material, which is available to authorized users.

## Background

Interspecies hybridization is a useful and important strategy for generating progeny with heterosis or hybrid vigor, which is a phenomenon that has been observed by naturalists, harnessed by breeders for centuries [1]. The utility of hybridization has tremendously increased the productivity in many species [2]. Preternaturally, through interspecies hybridization, natural polyploid hybrids are often produced.

Natural hybrid polyploidization plays an important role in eukaryote speciation and evolution, especially in plants and lower vertebrates [3–6]. Hybrid polyploids have been found in many types of fish and have achieved great heterosis, such as hybrids of *Carassius auratus* red var. × *Megalobrama amblycephala* [5, 7, 8], *Carassius auratus* red var*.* ♀ × *Erythroculter ilishaeformis* ♂ [9], and *Ctenopharyngodon idellus* ♀ × *Megalobrama amblycephala* ♂ [10]. Although superior performance of polyploid hybrids has been detected and the related breeding methods have been frequently applied, the genetic basis of heterosis or hybrid vigor is still unclear [1, 11]. Moreover, the polyploidization processes have not been characterized.

The 5S ribosomal DNA (5S rDNA) gene is an important and useful resource for analyzing and tracing rapid evolutionary events. In vertebrates, the 5S rDNA unit is formed by 120-bp highly conserved coding sequence and highly variable non-transcribed spacer (NTS) [5]. The high level conservation of 5S coding sequence arises from their essential molecular function [12, 13]. However, the NTS sequences show extensive variation, even between closely related species [13]. Many 5S rDNA studies had demonstrated the influence of polyploidy on intragenomic variation and gene expression [5, 14].

Our previous study had reported the generation of natural triploid hybrid groupers from the crossing of female *Epinephelus coioides* (*E. coioides*) and male *Epinephelus lanceolatus* (*E. lanceolatus*) [15]. The triploid hybrids had been proved to possess superior growth performance to that of the diploid hybrids and the parent species [15]. In the present study, fine karyotype analyses were performed to investigate the chromosome filiation of diploid and triploid hybrids, triploidization processes were traced based on embryo images and sections. The genetic basis of the hybrids was evaluated by 5S rDNA gene sequences. Chromosome location was carried out to further investigate the 5S rDNA gene pattern.

## Results

### Chromosomal origin

**Fig. 1:**
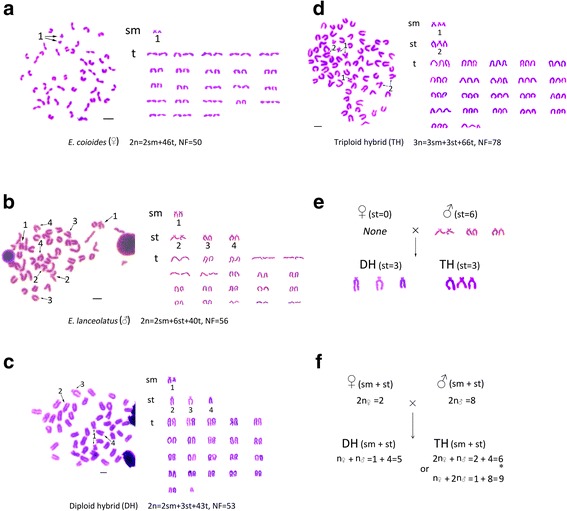
Karyotypic analysis of the groupers. **a** metaphase images of *E. coioides* (♀); **b** metaphase images of *E. lanceolatus* (♂); **c** metaphase images of diploid hybrid grouper (DH); **d** metaphase images of triploid hybrid grouper (TH); **e** inheritance relationship of the ‘st’ chromosomes; **f** quantity inheritance relationship of the two-armed chromosomes, ‘*’ indicated the true composition; sm: submetacentric chromosome; st: subtelocentric chromosome; t: telocentric chromosome; NF: number of the total fact arms. Arrows: the two-armed chromosomes of corresponding fish types. Bars = 5 μm

Fine karyotype analysis was conducted to infer chromosomal origins in the hybrids. Representative chromosome images of the four groupers were showed in Fig. 1; *E. coioides* had the karyotype formula 2n = 2sm + 46 t, NF = 50 (Fig. 1-a); *E. lanceolatus* had the karyotype formula 2n = 2sm + 6st + 40 t, NF = 56 (Fig. 1-b); the diploid hybrids had the karyotype formula 2n = 2sm + 3st + 43 t, NF = 53 (Fig. 1-c); and the triploid hybrids had the karyotype formula 3n = 3sm + 3st + 66 t, NF = 78 (Fig. 1-d).

The inheritance relationships of ‘st’ chromosome were displayed in Fig. 1-e; ‘st’ numbers of the groupers were 0, 6, 3, and 3 for *E. coioides*, *E. lanceolatus*, diploid hybrids, and triploid hybrids, respectively. The mother species, *E. coioides*, had no ‘st’ chromosome. The father species, *E. lanceolatus*, had 6 ‘st’ chromosomes. Both the diploid and triploid hybrids had the same ‘st’ numbers of 3, half of the father species, which indicated that the father species supplied one set of chromosomes to diploid and triploid hybrids respectively. Therefore, the mother species might supply one set of chromosomes to the diploid hybrids and two sets of chromosomes to the triploid hybrids.

The assumption that mother species supplied two sets of chromosomes to triploid hybrids was supported by the results shown in Fig. 1-f. As shown in Fig. 1, the 2n mother species had 2 two-armed chromosomes (Fig. 1-a), and the 2n father species had 8 two-armed chromosomes (Fig. 1-b). The diploid hybrid had inherited 1 two-armed chromosome from the mother species (half of 2) and 4 two-armed chromosomes from the father species (half of 8), the number of two-armed chromosomes in diploid hybrid was 5 (Fig. 1-c, f). The number of two-armed chromosomes in triploid hybrids might be 6 (2 + 4, two sets of mother species chromosomes plus with one set of father species chromosomes) or 9 (1 + 8, one set of mother species plus with two sets of father species) (Fig. 1-f), as shown in Fig. 1-d, the triploid hybrids had 6 two-armed chromosomes. These results indicated that triploid hybrids had two sets of chromosomes inherited from the mother species plus with one set of chromosomes inherited from the father species.

### Embryo development

**Fig. 2:**
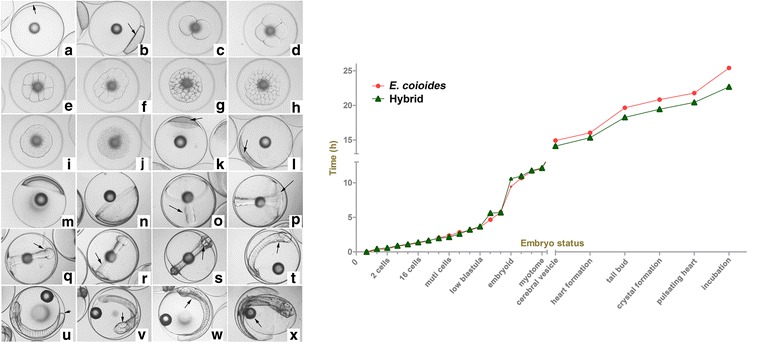
Embryo development in *E. coioides* and hybridization. The left images showed the embryo development processes of *E. coioides*. The right line chart indicated the hatch time of *E. coioides* and the hybrid. **a** unfertilized egg, arrow mean the unstimulated blastodisc; **b** blastodisc formation stage, the formation blastodisc as the arrow showed; **c** 2 cells stage; **d** 4 cells stage; **e** 8 cells stage; **f** 16 cells stage; **g** 32 cells stage; **h** 64 cells stage; **i** multi cells stage; **j** morula stage; **k** high blastula stage, arrow showed the heaving embryo; **l** low blastula stage, arrow showed the embryo; m: early gastrula stage; **n** mid gastrula stage; **o** embryoid formation stage; **p** blastopore closure stage, arrow showed the blastopore; **q** optic capsule formation stage, arrow showed the optic capsule; **r** myotome formation stage, arrow showed the mytotome; **s** cerebral vesicle formation stage, arrow showed the cerebral vesicle; **t** heart formation stage, arrow showed the heart; **u** tail bud stage, arrow showed the tail bud; **v** crystal formation stage, arrow showed the crystal; **w** heart beat stage, arrow showed the beating heart; **x** incubation stage, arrow showed the Oil globule. Magnification: 40 ×

To estimate the triploidization processes, embryo developments of the hybrids and *E. coioides* had been traced. The eggs were hatched in 27 °C, 30 ‰ salt seawater with sufficient air supplement. Images of the related development processes were obtained with a Nikon 50i camera microscope. Time consuming points were recorded. Representative images of *E. coioides* were showed in Fig. 2 (left). No substantial differences were found between images of the hybrid and the *E. coioides*. The consuming time points were exhibited in Fig. 2 (right) and Additional file 3: Table S1. From the line chart in Fig. 2 (right), hybrid embryos hatched 2–3 h earlier than those of *E. coioides*.

**Fig. 3:**
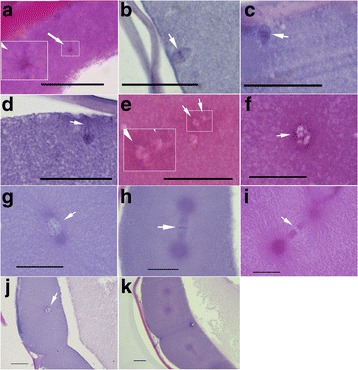
Histological sections of early embryo development of *E.coioides.* **a** 30 s after fertilization (AF), white arrows mean the sperm aster, which was framed in the white box and enlarged beside; **b** 1 min AF, white arrow indicated sister chromatids in the second meiosis metaphase stage; **c** 2 min AF, white arrow indicated the separating sister chromatids in the second meiosis anaphase; **d** 4 min AF, the second meiosis telophase, white arrow indicated the second polar nuclear; **e** 8 min AF, white arrows indicated the fusion of the two pronuclei, which were framed in the white box and enlarged beside; **f** 16 min AF, white arrow indicated the replication bubblo; **g** 20 min AF, white arrow indicated the replication bubblo; **h** 24 min AF, white arrow indicated metaphase chromosome; **i** 28 min AF, white arrow indicated the divorcing chromosomes; **j** 36 min AF, white arrow indicated the replication bubblo; **k** 40 min AF. Bars = 20 μm

To further survey the triploidization processes, histological sections of early embryo developments of the hybrid and *E. coioides* were carried out. Early embryo development processes of *E. coioides* were showed in Fig. 3. Spermaster was observed 30 s after fertilization (AF) (Fig. 3-a), the eggs remained in the second metaphase stage of meiosis at 1 min AF (Fig. 3-b), the eggs excluded the second polar body at 2–4 min AF (Fig. 3-c,d), the two pronuclei met and merged together at about 8 min AF (Fig. 3-e), the first cleavage took place at 24–28 min AF (Fig. 3-h-j).

**Fig. 4:**
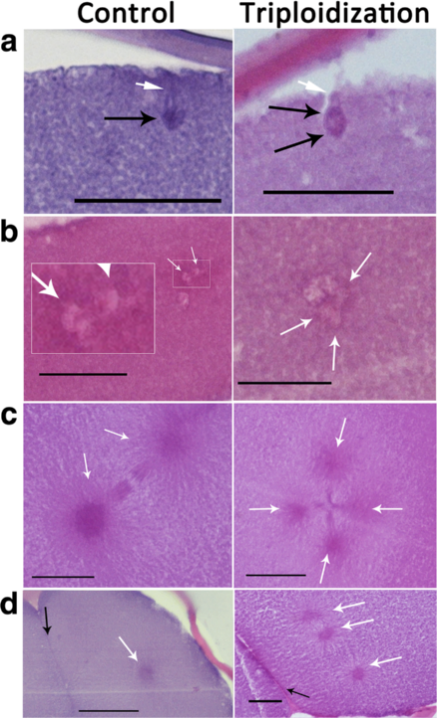
Triploidization processes in the hybrid*.* Control: embryo sections of *E. coioides*. Triploidization: triploidizition processes of the hybrid embryos. **a** the second meiosis telophase stage, white arrow in the control group indicated the formation of second polar body nucleus, white arrow in hybrid group indicated the excluding of the second polar body, the black arrows in this stage indicated the reserved pronuclei. **b** pronuclei fusion stage, white arrows in this stage indicated the combined pronuclei. **c** anaphase stage in the first cell division, white arrows in this stage indicated the spindle bodies. **d** telophase stage of the first cleavage, white arrows in this stage indicated the spindle bodies, *black arrows* in this stage indicated the cell plate. Bars = 20 μm

Triploidization processes in hybrid embryos were showed in Fig. 4. In control group of *E. coioides* specimens, normal polarized meiosis took place (Fig. 4-a), female and male pronuclei fused (Fig. 4-b), two spindle centers were observed in the anaphase stage in the first cell division (Fig. 4-c), only a spindle center was observed in the side of the cell plate after the first cleavage (Fig. 4-d). In the corresponding stages of hybrid embryos, abnormal phenomenon was observed during polarized meiosis (Fig. 4-a), the female pronucleus might retain two sets of genomes, a pseudo polar body without genome might be extruded; tri-polarized nucleus was observed (Fig. 4-b), two sets of maternal nuclei might fuse with the paternal pronucleus; four spindle centers were observed in the anaphase stage in the first cell division (Fig. 4-c); three spindle centers were observed in the side of the cell plate after the first cell cleavage (Fig. 4-d). These results indicated that triploidization processes might be occurred during the second meiosis stage in hybrid eggs, the first mitoses in the triploid embryos were different from that of *E. coioides*.

### 5S gene sequence analysis

**Fig. 5:**
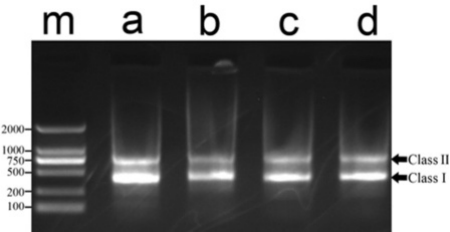
DNA bands of 5S rDNA gene of the groupers. **m**: DNA ladder marker (DS 2000, Dongsheng Company, china); (**a**)* E. coioides*; (**b**) *E. lanceolatus*; (**c**) diploid hybrid; (**d**) triploid hybrid. Class I and Class II were pointed out by arrows, the two bands were nearly 400 bp and 750 bp respectively

PCR amplifications of *E. coioides*, *E. lanceolatus*, diploid hybrids, and triploid hybrids were conducted and gel-separated. As showed in Fig. 5, all the lanes had two band patterns of approximately 400 bp (Class I) and 750 bp (Class II). To evaluate the 5S gene, a total of 160 clones were sequenced, including 15 clones of each band of each parental species and 25 clones of each band of each hybrid species. BLASTn was employed for sequence analyzing. Resulting data were summarized in Table 1. From Table 1, the Class I sequences contained the coding (120 bp) and NTS (266–284 bp) regions, which indicated that it was the monomeric structure of 5S gene. Compared with the Class I in parental species, the hybrids had no novel sequences. The Class II sequences were detected to contain the dimeric Class I structures, whereas, novel sequences were detected (781 bp, 790 bp) in diploid and triploid hybrids. The 781 bp sequences were the combination structure of 386 bp (maternal Class I) and 395 bp (paternal Class I), the 790 bp sequences were the combination structure of 386 bp (maternal Class I) and 404 bp (paternal Class I). These results indicated that the new sequences in the hybrids were combined with maternal and paternal monomeric sequences, the tandemly repeated units of the parental 5S rDNA genes might exchange with each other during the hybridization.

**Table 1:** Gene structure of different classes of 5S rDNA

	400 bp (Class I)			750 bp (Class II)		
	Sequences	Numbers	Structure	Sequences	Numbers	Structure
*E. coioides*	386 bp	9	120 bp + 266 bp	772 bp	7	386 bp + 386 bp
	392 bp	6	120 bp + 272 bp	778 bp	4	386 bp + 392 bp
				784 bp	4	392 bp + 392 bp
*E. lanceolatus*	395 bp	5	120 bp + 275 bp	790 bp	5	395 bp + 395 bp
	404 bp	10	120 bp + 284 bp	799 bp	3	395 bp + 404 bp
				808 bp	7	404 bp + 404 bp
Diploid hybrid	386 bp	7	120 bp + 266 bp	772 bp	6	386 bp + 386 bp
	392 bp	4	120 bp + 272 bp	781 bp^a^	3	386 bp + 395 bp^b^
	395 bp	6	120 bp + 275 bp	790 bp^a^	6	386 bp + 404 bp^b^
	404 bp	8	120 bp + 284 bp	799 bp	3	395 bp + 404 bp
				808 bp	7	404 bp + 404 bp
Triploid hybrid	386 bp	10	120 bp + 266 bp	772 bp	9	386 bp + 386 bp
	392 bp	3	120 bp + 272 bp	781 bp^a^	2	386 bp + 395 bp^b^
	395 bp	4	120 bp + 275 bp	790 bp^a^	2	386 bp + 404 bp^b^
	404 bp	8	120 bp + 284 bp	799 bp	3	395 bp + 404 bp
				808 bp	9	404 bp + 404 bp

^a^New sequences which were different from the parent species

^b^Gene-exchange had occurred in the sequence

**Fig. 6:**
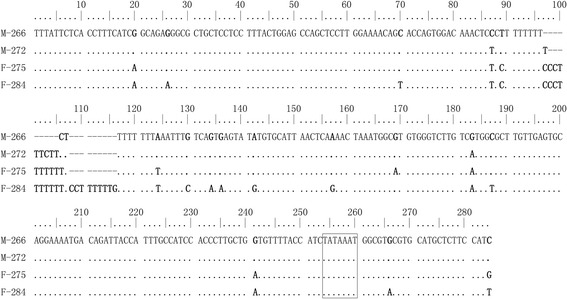
Alignment results of 5S gene NTS sequences from parental species. M-266, 272: 266 bp and 272 bp of NTS sequences from 386 bp and 392 bp complete 5S genes of mother species (M) (*E. coioides*), respectively; F-275, 284: 2275 bp and 284 bp of NTS sequences from 395 bp and 404 bp complete 5S genes of father species (M) (*E. lanceolatus*), respectively. Dots indicated the identical nucleotides, hyphens represented the insertions or deletions. In bold letters were shown the nucleotide substitutions. The TATA sequences were framed in boxes

Multiple analysis of the 5S coding region had been conducted. The results were showed in Additional file 1: Figure S1. There were five variable positions in the aligned sequences (positions 64, 68, 69, 82, and 106). Alignment results of the NTS sequences were listed in Additional file 2: Figure S2. There were total 4 variable positions in the 266-bp NTS sequence alignments, 11 in the 272-bp NTS sequence alignments, 13 in 275-bp NTS sequence alignments, and 15 in the 284-bp NTS sequence alignments. ‘TATA’-like promoter elements were found 30 bp upstream of the ‘ATG’ promoter. The sequences of the ‘TATA’ box in the four groupers were ‘TATAAAT’. Parental NTS sequences (M-266, M-272, F-275, F-284) were aligned, the results were showed in Fig. 6. There were 36 different (variable or absent) positions. The most diverse sequences were found in M-266 (from *E. coioides*, 386 bp, M-386) and F-284 (from *E. lanceolatus*, 404 bp, F-404), they had 16 variable and 19 absent positions.

### Chromosomal location of the 5S gene

**Fig. 7:**
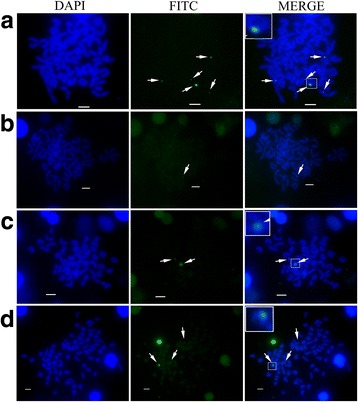
Chromosome location of 5S rDNA gene in the groupers. **a** The staining images of *E. coioides*; **b** The staining images of *E. lanceolatus*; **c** The staining images of diploid hybrid; **d** The staining images of triploid hybrid. Chromosomes were stained with DAPI, signals were detected by FITC-conjugated antibody. The white arrows indicated the fluorescent signals (*green loci*). The strong signals were framed and partial enlarged in the white boxes. Bars = 5 μm

Triploid hybrid groupers had two sets of maternal chromosomes. The M-386 sequence (GenBank Accession No. KR262163) was used as the probe. Results were listed in Table 2 and Fig. 7. Based on the information in Table 2, the chromosome loci numbers of *E. coioides*, *E. lanceolatus*, diploid hybrids, and triploid hybrids were mostly 5, 1, 2, and 3, respectively. Representative pictures of 5S gene chromosomal location were displayed in Fig. 7. There were fewer fluorescence loci in *E. lanceolatus* metaphase chromosomes (Fig. 7-b) than that in *E. coioides* (Fig. 7-a), and signal intensity of *E. lanceolatus* was weaker than that of *E. coioides*. More fluorescence loci numbers were detected in triploid hybrids than that in diploid hybrids and *E. lanceolatus*. Strong signals (in the white frames of Fig. 7-a, c, d) were found in *E. coioides*, diploid hybrids, and triploid hybrids. The strong signals were in the middle of chromosome in diploid hybrids, whereas, the signals were in the upper end of the chromosome in *E. coioides* and triploid hybrids.

**Table 2:** Chromosome fluorescence signal numbers of the four groupers

			Distribution of chromosome loci numbers				
Fish type	Individuals	Metaphase numbers	1	2	3	4	5
*E. coioides*	5	100	3	7	11	19	60
*E. lanceolatus*	5	100	86	12	2	0	0
Diploid hybrid	5	100	17	74	7	2	0
Triploid hybrid	5	100	9	13	62	14	2

## Discussion

Karyotype analysis is a useful method to trace chromosome filiations in polyploid hybrid progenies. Karyotype analyses by Qin et al. (2014) and He et al. (2012) indicated that triploid hybrids had two sets of chromosomes from the mother species and one set from the father species, tetraploid hybrids had two sets of chromosomes from the mother and two sets from the father [8, 9]. In this paper, triploid hybrid groupers were produced from *E. coioides* ♀ × *E. lanceolatus* ♂, karyotype results showed that triploid hybrids had two sets of chromosomes from the mother species and one set from the father species.

Natural polyploids were more and more commonly detected in many fish species in recent years. Tsuda et al. (2010) detected the occurring of triploid *Rhamdia quelen* [16]. Bai et al. (2011) reported the coexisting populations of diploid, triploid and polyploidy *Carassius auratus* individuals [17]. Triploid hybrids were generated from the interspecies hybridization of female *Ctenopharyngodon idellus* and male *Megalobrama amblycephala* [10]. However, the cellular formation processes of the polyploids were still unclear. In this study, embryo images and sections were conducted to investigate the triploidization processes. Results showed that the hybrid embryo images were not substantially different from the related of *E. coioides*, but the hybrid embryo had the abnormal polarized meiosis and cell division processes. The hybrid female pronucleus might retain two sets of chromosomes and extrude a pseudo polar body without genome. To our knowledge, this was the first report of cellular polyploidization processes in interspecies hybridization. Our results suggested that the extra sets of chromosomes in triploids might be caused by failure formation of the second polarized genome.

Analysis of genetic organization and variation may be helpful in evaluating the role of hybridization. The NTS of 5S rDNA gene is neutral and freely mutative, and it has been widely employed as a molecular marker to assess the organization of genomes and to trace recent evolutionary events [13, 18]. Analysis of different ploidy hybrids of *Carassius auratus* red var*.* ♀ and *Megalobrama amblycephala* ♂ showed that the tetraploid hybrids partially inherited NTS classes from the female parent and obtained a novel NTS sequence [5]; similar results were obtained from tetraploid hybrids of *Carassius auratus* red var*.* ♀ and *Erythroculter ilishaeformis* ♂ [9]. In the present study, the 5S rDNA genes were cloned and analyzed, Class II sequences (781 bp, 790 bp) in diploid and triploid hybrids were composed of two monomer structures (Class I) which were originated from their parent species. The results indicated that the new sequences in the hybrids were recombined by the parental sequences. In this paper, the NTS sequences in diploid and triploid hybrids were slightly different, but not blocks of variant sequences, the results were similar to that of He et al. (2013) [10], the chimera sequences (except for a 10-bp poly A insertion in diploid hybrids) were found in the diploid and triploid hybrids [10]. The number of fluorescent loci in the triploid hybrids was less than that in mother species, which indicated that the recombination of 5S gene might enfeeble the efficiency of M-386 probe in triploid hybrid chromosomes.

However, despite this comprehensive investigation, a general and basic understanding of heterosis remains elusive. Single loci have a relatively small contribution to hybrid traits [2]. Heterosis or hybrid vigor may be shaped by different traits that are affected by multiple gene loci [19]. In our study, the new 5S gene sequences in diploid and triploid hybrids were composed of different parental monomer structures. The results indicated that genome loci of parental species might be exchanged or recombined during the hybridization. Our results would provide the basic molecular evidence for explaining the heterosis phenomenon in hybrids.

## Conclusions

In conclusion, triploidization processes and genetic patterns of 5S rDNA in hybrid groupers had been investigated in this paper. The extra sets of chromosomes in triploid hybrids had been demonstrated to come from the mother species. The failure exclusion of sister chromosomes might be occurred during the second meiosis stage, a pseudo polar body without genome might be excluded instead. Hybrid heterosis might be induced by interspecies genome re-fusion and gene exchanging. More evidence and further research are still needed to reveal the molecular mechanisms of natural polyploidization and hybrid vigor.

## Methods

### Karyotype analysis

Karyotype analyses of each five individuals of *E. coioides* (the female parent), *E. lanceolatus* (the male parent), diploid hybrids, and triploid hybrids were operated to infer the chromosomal origin. Karyotype slides were prepared from cultured peripheral blood cells. Detailed processes were conducted as described by Huang et al. (2014) [15]; blood was collected and cultured in nutrient solution at 25.5 °C with 5 % CO_2_ for 68–72 h, and 0.1 ml colchicines solution was added 3 h before harvest. The cells were then treated with 0.075 M KCl at 37 °C for 30 min. Blood cells were dropped on the cold slides and air-dried at room temperature. Chromosomes were stained by Giemsa solution (pH 6.8) for 30 min, washed in running water, air-dried. Good-quality metaphase spreads images were obtained by a Nikon 50i microscope (Nikon, Japan) for karyotype analyzing. Fine karyotype analysis was carried out as Qin et al. (2014) [8] had described. Long-arm to short-arm ratios of 1.0–1.7 were classified as metacentric (m), 1.7–3.0 as submetacentric (sm), 3.1–7.0 as subtelocentric (st), and > 7.1 as telocentric (t) chromosomes (Levan et al. 1964) [20﻿].

### Embryo development

Breeding of *E. coioides* and hybridization of *E. coioides* ♀ × *E. lanceolatus* ♂ were carried out. The methods were conducted as described in Huang et al. (2014) [15]. Good-quality eggs were hatched in seawater (nearly 30‰ salinity) at room temperature. The embryo development processes were imaged by a Nikon 50i microscope camera. The fry were reared to fingerlings in the same environment.

Eggs were fixed in Bouin’s solution after fertilization of 30 s, 1 min, 2 min, 3 min, 4 min, 6 min, and 8 min and then every 4 min until 40 min. The eggs were dehydrated in alcohol, embedded in paraffin wax, sectioned, and stained with hematoxylin and eosin. Photomicrographs were taken with a Nikon 50i microscope camera.

### 5S rDNA genetic analysis

Total genomic DNA of *E. coioides* (the female parent), *E. lanceolatus* (the male parent), diploid hybrids, and triploid hybrids were extracted from the caudal fin with a TIANamp Marine Animals DNA Kit (TIANGEN, China). The primers of 5S-F (5′-GCTTACGGCCATACCAACCTG-3′) and 5S-R (5′-AATACGCCCGATCTCGTCCGA-3′) were designed and synthesized to amplify the 5S rDNA gene. PCR conditions were the same as described by Qin et al. (2010) [5]. Amplification products were gel-separated and purified with an EZNA® Gel Extraction Kit (Omega, United States). The purified DNA products were ligated into the pMD18-T vector (TaKaRa, China) and transformed into *E. coli* DH5a. Monoclonal bacteria were obtained from culturing *E. coli* DH5a. Thirty monoclonal bacteria of parent grouper species and fifty monoclonal bacteria of each hybrid species were sequenced with an automated DNA sequence (ABI PRISM 3730, Applied Biosystems, United States). Sequences were analyzed with ClustalW 2.0 and Bioedit 7.2.

### Chromosomal location of 5S rDNA

The chromosomal locations of the 5S rDNA gene were conducted in *E. coioides* (the female parent), *E. lanceolatus* (the male parent), diploid hybrids, and triploid hybrids. Probes labeled with DIG-11-dUTP (Roche, Germany) were made by purifying the PCR products of 5S rDNA. Fluorescence in situ hybridization was performed as described by He et al. (2012) [9]. Images were obtained and merged by the NIS-Elements software of Nikon 50i fluorescence microscope system (Nikon, Japan). For each type of grouper, 100 metaphase spreads (20 metaphase spreads per sample) of chromosomes were analyzed.

## Additional files

Additional file 1: Figure S1. Alignment results of coding region of 5S gene. M-386, 392: coding region of 386 and 392 bp 5S sequences from mother species (*E. coioides*); F-404, 395: coding region of 404 and 395 bp 5S sequences from father species (*E. lanceolatus*); D-404, 395, 392, 386: coding region of 404, 395, 392 and 386 bp 5S sequences from diploid hybrid; T-404, 395, 392, 386: coding region of 404, 395, 392 and 386 bp 5S sequences from triploid hybrid. Dots indicated the identical nucleotides. The Internal Control Region (A box: positon 50–64 bp, internal element: position 67–72 bp, C box: position 80–97 bp) were framed in the boxes. (TIF 979 kb)
Additional file 2: Figure S2. Alignment results of 5S gene NTS sequences from all kinds of groupers. a: The 266 bp NTS sequences of *E. coioides*, diploid hybrid and triploid hybrid; b: The 272 bp NTS sequences of *E. coioides*, diploid hybrid and triploid hybrid; c: The 275 bp NTS sequences of *E. lanceolatus*, diploid hybrid and triploid hybrid; d: The 284 bp NTS sequences of *E. lanceolatus*, diploid hybrid and triploid hybrid. The TATA sequences were framed in boxes. Dots indicated the identical nucleotides. In bold letters were shown the nucleotide substitutions. (TIF 2265 kb)
Additional file 3: Table S1. Embryo development records of the groupers. (XLSX 13 kb)

## Abbreviations

5S rDNA or 5S gene: 5S ribosomal DNA gene
AF: After fertilization
D-404, 395, 392, 386: Coding region of 404, 395, 392 and 386 bp 5S sequences from diploid hybrid
*E. coioides*: *Epinephelus coioides*
*E. lanceolatus*: *Epinephelus lanceolatus*
F-404, 395: Coding region of 404 and 395 bp 5S sequences from father species (*E. lanceolatus*)
M-386, 392: Coding region of 386 and 392 bp 5S sequences from mother species (*E. coioides*)
NTS: Non-transcribed spacer
T-404, 395, 392, 386: Coding region of 404, 395, 392 and 386 bp 5S sequences from triploid hybrid
