# Supplementary figures and images for: Formation of diploid and triploid hybrid groupers (hybridization of Epinephelus coioides ♀ × Epinephelus lanceolatus ♂) and their 5S gene analysis
Source: BMC Genet. 2016 Oct 7;17:136. doi: 10.1186/s12863-016-0443-9 (PMC5054551; doi:10.1186/s12863-016-0443-9)

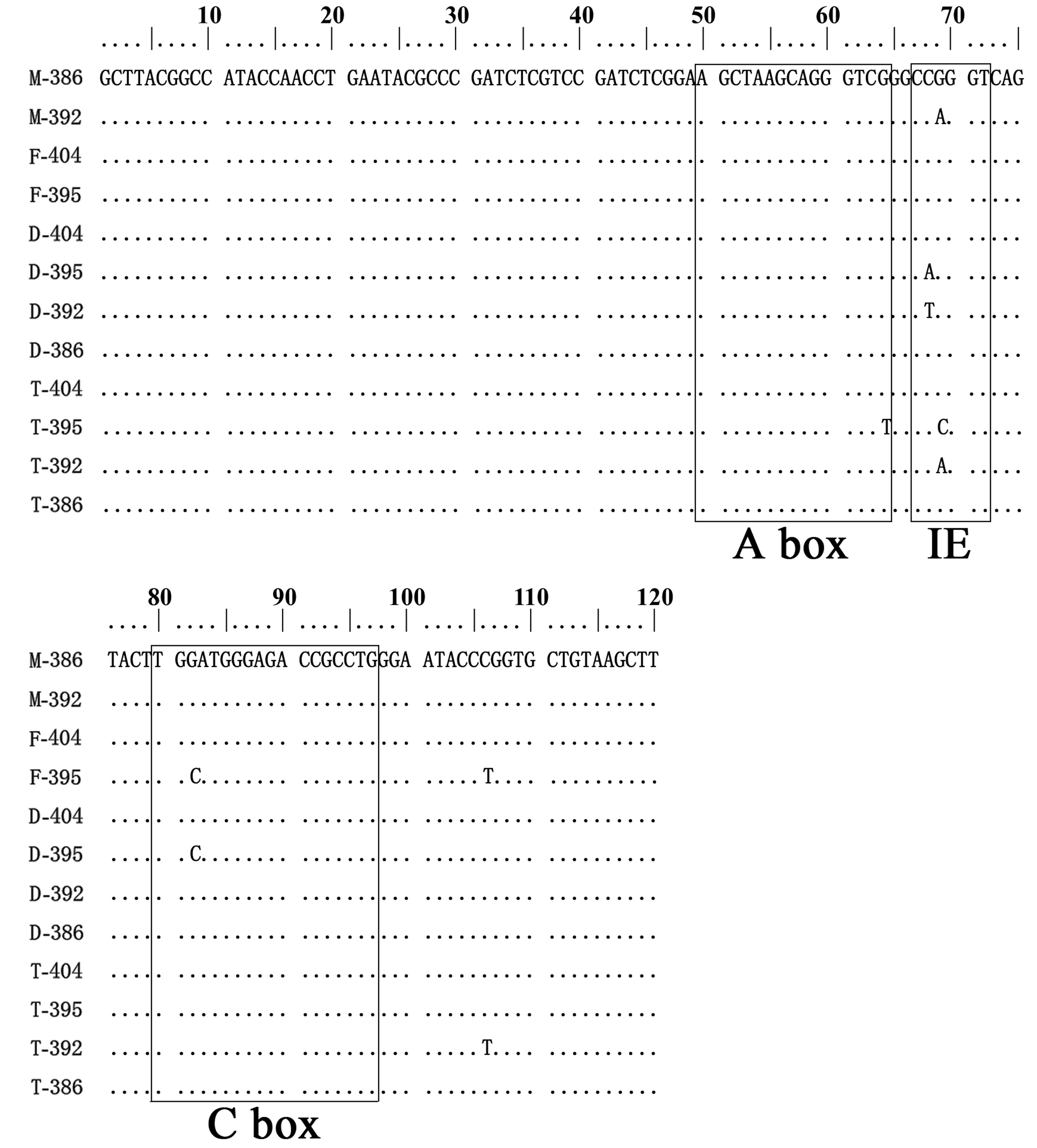

Supplement: Additional file 1: Figure S1. — Alignment results of coding region of 5S gene. M-386, 392: coding region of 386 and 392 bp 5S sequences from mother species (E. coioides); F-404, 395: coding region of 404 and 395 bp 5S sequences from father species (E. lanceolatus); D-404, 395, 392, 386: coding region of 404, 395, 392 and 386 bp 5S sequences from diploid hybrid; T-404, 395, 392, 386: coding region of 404, 395, 392 and 386 bp 5S sequences from triploid hybrid. Dots indicated the identical nucleotides. The Internal Control Region (A box: positon 50–64 bp, internal element: position 67–72 bp, C box: position 80–97 bp) were framed in the boxes. (TIF 979 kb) [file 12863_2016_443_MOESM1_ESM.tif]

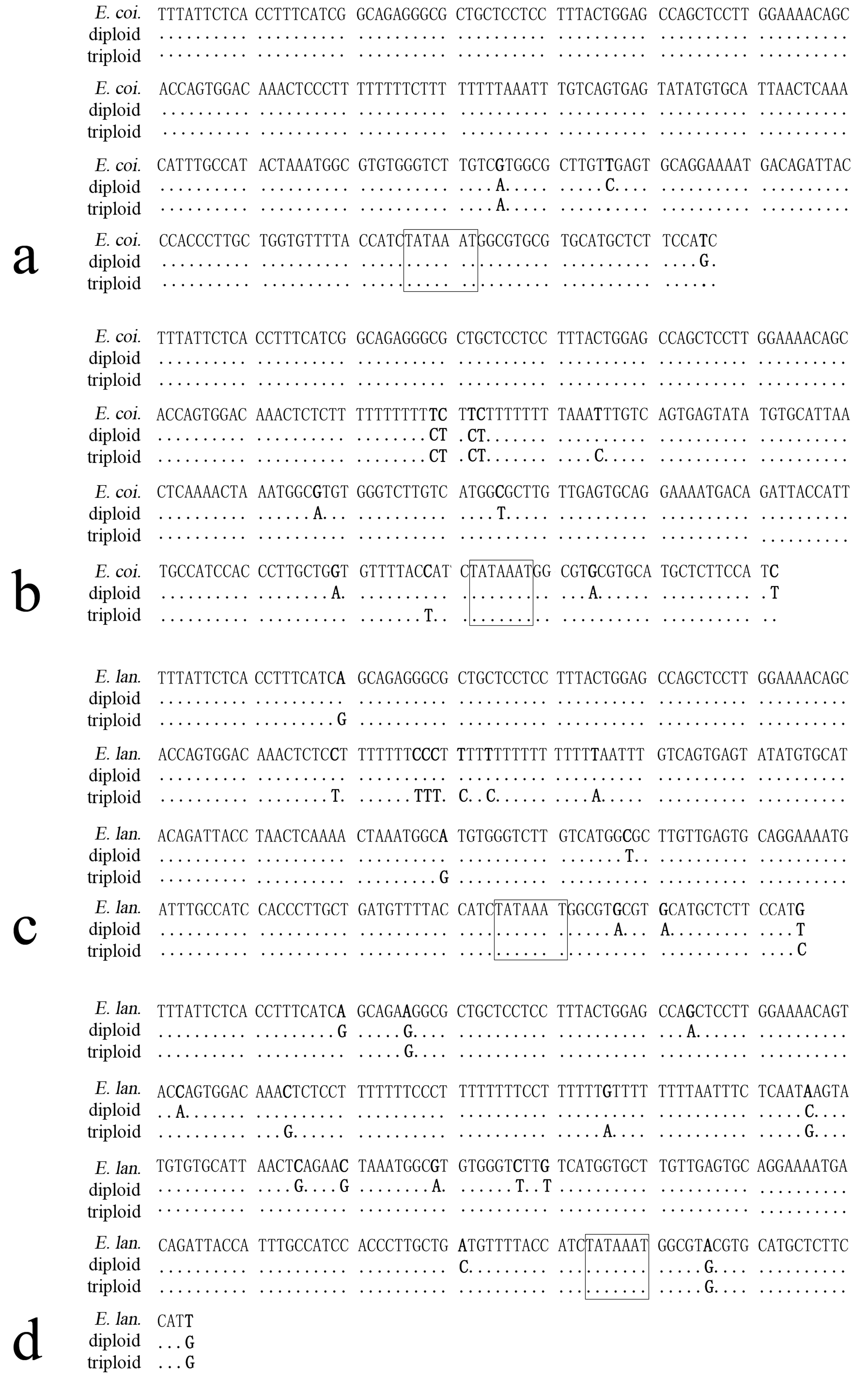

Supplement: Additional file 2: Figure S2. — Alignment results of 5S gene NTS sequences from all kinds of groupers. a: The 266 bp NTS sequences of E. coioides, diploid hybrid and triploid hybrid; b: The 272 bp NTS sequences of E. coioides, diploid hybrid and triploid hybrid; c: The 275 bp NTS sequences of E. lanceolatus, diploid hybrid and triploid hybrid; d: The 284 bp NTS sequences of E. lanceolatus, diploid hybrid and triploid hybrid. The TATA sequences were framed in boxes. Dots indicated the identical nucleotides. In bold letters were shown the nucleotide substitutions. (TIF 2265 kb) [file 12863_2016_443_MOESM2_ESM.tif]
